# Screening and Identification of Antioxidant Peptides from Sea Cucumber Gonad Proteins and Their Activation of Superoxide Dismutase

**DOI:** 10.3390/foods14223848

**Published:** 2025-11-11

**Authors:** Zhiqin Zhang, Jingxuan Wang, Yongke Deng, Yugui Wang, Peipei Dou, Hongbing Fan, Xiangquan Zeng, Xinguang Fan, Lili Zhang, Haimei Liu, Qin Zhao

**Affiliations:** 1 College of Food Engineering, Ludong University, Yantai 264025, China; zhangzhiq924@163.com (Z.Z.); 2024121073@m.ldu.edu.cn (J.W.); 2024121064@m.ldu.edu.cn (Y.D.); firelighting@126.com (X.F.); lilizhang1126@126.com (L.Z.); 2 Department of Animal and Food Sciences, University of Kentucky, Lexington, KY 40546, USA; yugui.wang@uky.edu (Y.W.); peipei_dou@uky.edu (P.D.); hongbing.fan@uky.edu (H.F.); 3 Department of Food Science, College of Agriculture, Purdue University, West Lafayette, IN 47907, USA; 20210803@btbu.edu.cn

**Keywords:** sea cucumber gonad, antioxidant peptide, virtual screening, molecular docking, molecular dynamics simulation

## Abstract

The gonad is one of the major byproducts of sea cucumber. Four novel antioxidant peptides (NPWGQ, PGHPF, VPYPR and ATGPQGPAGQRGPAGPTGPTGPAG) were isolated and identified from sea cucumber gonad proteins through enzymatic hydrolysis and antioxidant activity-guided fractionation, bioinformatics approaches and in silico screening. These peptides demonstrated great free radical (2,2-diphenyl-1-picrylhydrazyl (DPPH) and 2,2′-azinobis(3-ethylbenzothiazoline-6-sulfonic acid) (ABTS))-scavenging activity and notable superoxide dismutase (SOD)-activation capacity. Molecular docking and molecular dynamics simulation data suggested that these peptides could form strong binding with SOD through hydrogen bonding, electrostatic interactions, and hydrophobic interactions. Among these peptides, NPWGQ displayed the most potent antioxidant and SOD-activating effects. Through searching known databases, these peptides did not show potential toxicity and are generally considered safe. The present study provides crucial theoretical support for comprehensively utilizing sea cucumber (*Holothuroidea*) gonad by-products and generating high-value functional food ingredients or dietary supplements.

## 1. Introduction

Oxidative stress is one of the key causative factors of various chronic diseases, including cardiovascular diseases, neurodegenerative diseases, and cancer. Its central mechanism involves cellular and tissue damage caused by excessive accumulation of free radicals [1,2]. Although common synthetic antioxidants such as butylated hydroxyanisole (BHA) and butylated hydroxytoluene (BHT) exhibit certain inhibitory effects on oxidative processes, studies suggest these compounds pose potential health risks [3]. In recent years, natural antioxidants have gained prominence due to their safety and efficacy. Among these, antioxidant peptides stand out for their unique bioactivity, low toxicity, high safety, and tolerance [4].

Sea cucumbers (*Holothuroidea*), belonging to the phylum Echinodermata, are highly valued marine delicacies, particularly in Asian countries such as China, Japan, and Korea. Global sea cucumber production has surged in recent years, with China alone producing over 200,000 tons annually, driven by increasing consumer demand due to their nutritional and medicinal benefits [5]. Sea cucumbers are rich in proteins, amino acids, polysaccharides, saponins, and essential macro- and microelements, making them a prized functional food in traditional Chinese medicine and gourmet cuisine. The sea cucumber body wall serves as the primary edible portion, resulting in substantial byproducts (e.g., gonadal tissues) being discarded during processing [6]. One major byproduct is gonads, which are mature reproductive organs during the spawning season and nutritionally valuable components of the viscera, containing abundant proteins, all eight essential amino acids, and high levels of unsaturated fatty acids. Recent advancements in sea cucumber body wall protein research have led to the isolation and identification of bioactive peptides with diverse physiological functions, including antioxidant [7], hypoglycemic [8], anti-inflammatory [9], and antitumor activities [10]. However, studies on antioxidant peptides derived from sea cucumber gonads remain relatively limited, and their antioxidant mechanisms and structure–function relationships have not yet been fully elucidated.

This study focuses on sea cucumber gonads, a major by-product generated during processing, with the aim of systematically exploring novel antioxidant peptides from this source. It seeks to address the challenge of waste disposal in the sea cucumber processing industry and promote the conversion of by-products into high-value functional ingredients, thereby achieving the sustainable goal of “turning waste into treasure.” The development of antioxidant peptides derived from sea cucumber gonads will not only expand the research scope of marine bioactive peptides but also open new pathways for the high-value utilization of marine biological resources.

Antioxidant peptides exert their antioxidant activities via multiple mechanisms. They can function as direct radical scavengers to neutralize free radicals [11,12] and suppress Nicotinamide Adenine Dinucleotide Phosphate—Hydrogen (NADPH) oxidase activity to reduce the generation of reactive oxygen species in mitochondria. They also activate the nuclear factor erythroid 2-related factor 2-Kelch-like ECH-associated protein 1 (Nrf2-Keap1) signaling pathway by facilitating Nrf2 dissociation from Keap1, thereby inducing the expression of antioxidant enzyme genes [13]. This process enhances the activity of key antioxidant or detoxifying enzymes including superoxide dismutase (SOD), catalase (CAT), and glutathione peroxidase (GPx), counteracting oxidative stress and maintaining cellular redox homeostasis [14]. Molecular docking and molecular dynamics (MD) simulation, as core technologies of computer-aided drug design (CADD) [15], provide robust tools for the efficient screening and mechanistic investigation of antioxidant peptides. Molecular docking predicts the binding modes and affinity between ligands, e.g., peptides and target receptors, e.g., proteins, enabling rapid identification of potential bioactive candidates. Molecular dynamics simulation further elucidates dynamic interactions, binding stability, conformational changes, and critical binding sites, offering theoretical foundations for understanding antioxidant mechanisms. Molecular docking has proven effective in studying the binding of antioxidant peptides to Keap1, a crucial regulator of the Nrf2-dependent antioxidant pathway [16]. MD simulation complements molecular docking by revealing the dynamic stability of the peptide-Keap1 complex and conformational changes that release Nrf2. Activated Nrf2 regulates the expression of a suite of antioxidant factors, including SOD, CAT, and GPx, thereby eliciting its antioxidant effects [17,18,19]. Antioxidant peptides have also been reported to interact with these enzymes directly, especially SOD. A dry-cured beef-derived peptide, Phe-Asp-Gly-Asp-Phe (FDGDF), could improve SOD activity by changing its secondary structure and exhibit its antioxidant capacity [20]. This raises our interest in exploring the interaction between antioxidant peptides and SOD and discovering more novel antioxidant peptides.

This study aims to develop novel antioxidant peptides from sea cucumber gonads through a combined approach of traditional fractionation based on free radical scavenging (2,2-diphenyl-1-picrylhydrazyl (DPPH) and 2,2′-azinobis(3-ethylbenzothiazoline-6-sulfonic acid) (ABTS)) assays and superoxide dismutase (SOD) activation assays, alongside bioinformatics methods (in silico screening). Subsequently, molecular docking and molecular dynamics simulation techniques will be employed to thoroughly investigate the interaction patterns between the identified active peptides and SOD. The focus will be on elucidating whether these peptides can directly bind to and modulate the conformation and activity of SOD, thereby synergistically enhancing antioxidant effects through dual pathways: “direct free radical scavenging” and “activation of endogenous antioxidant enzymes.”

## 2. Materials and Methods

### 2.1. Materials and Chemicals

Fresh sea cucumber gonads were purchased from Zhonglu Trading Co., Ltd. (Yantai, China), homogenized, and stored at −18 °C for further use.

Pepsin (from porcine gastric mucosa) was obtained from Sangon Biotech Co., Ltd. (Shanghai, China). Flavourzyme^®^ (from Aspergillus oryzae) was supplied by Solarbio Science & Technology Co., Ltd. (Beijing, China). Salicylic acid (analytical grade), 2,20-diphenyl-1-picrylhydrazyl (DPPH), 2,20-azinobis(3-ethylbenzothiazo-line-6-sulfonic acid) diammonium salt (ABTS), potassium persulfate, and reduced glutathione (GSH) were purchased from Sigma-Aldrich (Shanghai, China), TCI Chemicals (Shanghai, China), and Yuanye Bio-Technology Co., Ltd. (Shanghai, China), respectively. Ethanol (analytical grade) was provided by Sinopharm Chemical Reagent Co., Ltd. (Shanghai, China). The polypeptide was chemically synthesized by GenScript Biotech Corporation (Jiangsu, China), with a purity of ≥98% as verified by HPLC analysis.

### 2.2. Preparation and Fractionation of Enzymatic Hydrolysates from Sea Cucumber Gonads

The defatting process was performed according to the method described by [21] with modifications. Homogenized sea cucumber gonads were mixed with isopropanol at a 1:4 (*w*/*v*) ratio and incubated at 35 ± 2 °C for 1 h to remove lipids. The supernatant was discarded, and the residue was further defatted under the same conditions (35 ± 2 °C, 1:4 *w*/*v*) for 90 min.

The defatted sea cucumber gonad samples were mixed with deionized water at a ratio of 1:5 (g/mL), heated at 95 °C for 20 min, and cooled. The pH was adjusted to 2.5, followed by the addition of pepsin (2000 U/g) for enzymatic hydrolysis at 37 °C for 4 h. Subsequently, the pH was adjusted to 7.0, and flavourzyme (1200 U/g) was added for further hydrolysis at 50 °C for 2 h. The reaction was terminated by boiling at 100 °C, and the mixture was centrifuged at 5000 r/min for 15 min. The supernatant was collected as the enzymatic hydrolysate.

The hydrolysate was fractionated using spiral-wound membranes with molecular weight cut-offs (MWCO) of 200 Da and 3000 Da. Two peptide fractions were obtained: U1 (200–3000 Da) via sequential nanofiltration and ultrafiltration, and U2 (>3000 Da). All fractions were freeze-dried and stored at −18 °C for further analysis.

### 2.3. Determination of Molecular Weight Distribution of Peptide Fractions

The molecular weight distribution of peptide fractions was analyzed using high-performance liquid chromatography (HPLC, model, manufacturer) with minor modifications according to the method described by [22]. A calibration curve was constructed by plotting the logarithm of molecular weights against retention times, using the following standards: cytochrome C (12,365 Da), aprotinin (6511 Da), bacitracin (1450 Da), oxidized glutathione (612 Da), reduced glutathione (307 Da), and L-tryptophan (204 Da). The resulting regression equation was Y = −0.199X + 6.5188 (R^2^ = 0.9976), where Y represents the logarithm of the molecular weight and X corresponds to the retention time.

Chromatographic separation was performed on a TSK-GEL G2000SWXL column (7.8 mm × 300 mm) with an isocratic mobile phase consisting of acetonitrile–water–trifluoroacetic acid (20:80:0.1, *v*/*v*/*v*) at a flow rate of 0.5 mL/min. Detection was carried out at 220 nm, with the column maintained at 25 °C. Samples (10 μL) were filtered through a 0.22 μm membrane prior to injection. The molecular weight distribution of the peptide fractions was determined by comparing their retention times with the calibration curve.

### 2.4. Antioxidant Assays

#### 2.4.1. DPPH Free Radical Scavenging Assay

The DPPH free radical scavenging activity of sea cucumber gonad peptide fractions was determined according to the method of [23] with minor modifications. Briefly, peptide fractions were dissolved in deionized water at different concentrations, and a fresh 0.2 mmol/L DPPH solution was prepared in absolute ethanol (99%). For the assay, 100 μL of each sample was mixed with 100 μL of DPPH solution in 96-well plates (test group). The blank control contained deionized water instead of sample, a control group was prepared by mixing 100 μL of sample with 100 μL of absolute ethanol (without DPPH). After incubation in the dark at 25 °C for 30 min, the absorbance was measured at 517 nm. The DPPH radical scavenging activity was then calculated using the appropriate formula.

DPPH free radical scavenging rate (%) = [1 − (A_1_ − A_2_)/A_0_] × 100 (1)

where A_0_, A_1_ and A_2_ were the absorbance of the blank group, the sample group, and the control group.

#### 2.4.2. ABTS Radical Scavenging Assay

The ABTS radical scavenging activity was measured according to the methods of [24,25] with slight modifications. Briefly, ABTS stock solution was prepared by mixing 14 mmol/L ABTS and 4.95 mmol/L potassium persulfate, followed by incubation in the dark at room temperature for 12–16 h. Prior to use, the stock solution was diluted with deionized water to achieve an absorbance of 0.70 ± 0.02 at 734 nm (working solution).

For the assay, 150 μL of ABTS working solution was mixed with 50 μL of peptide solution at varying concentrations. After 60 s of reaction, the absorbance was measured at 734 nm using a microplate reader. The ABTS radical scavenging activity was calculated as follows:

ABTS free radical scavenging rate (%) = [1 − (A_1_ − A_2_)/(A_3_ − A_4_)] × 100 (2)

where A_1_, A_2_, A_3_, and A_4_ were the absorbance of the sample group, the sample background group, the blank group and the solvent background group.

#### 2.4.3. SOD Activation Assay

The selected groups were prepared as solutions (1–5 mg/mL), centrifuged, and filtered through a 0.45 μm membrane prior to analysis. The autoxidation rate of pyrogallol was determined based on a modified method of [26] with the rate controlled at 0.07 (± 0.002) OD/min. For sample measurement, Tris-HCl buffer, the test sample, and SOD solution were mixed and incubated at 25 °C for 10 min. Pyrogallol solution was then added, thoroughly mixed, and timed immediately. Absorbance was recorded at 325 nm at 30 s intervals for 3 min.

### 2.5. Peptide Sequence Identification and Virtual Screening

#### 2.5.1. Identification and Sequencing of Peptides by Liquid Chromatography–Tandem Mass Spectrometry

For each sample, an appropriate amount of peptides was separated using a nanoflow Easy nLC 1200 chromatography system (Thermo Scientific, Waltham, MA, USA). The mobile phases consisted of buffer A (0.1% formic acid in water) and buffer B (0.1% formic acid in acetonitrile/water mixture with 80% acetonitrile). The analytical column was equilibrated with 100% buffer A. After loading onto a Trap Column (100 µm × 20 mm, 5 µm, C18, Dr. Maisch GmbH, Ammerbuch, Germany), the peptides were separated on an analytical column (75 µm × 150 mm, 3 µm, C18, Dr. Maisch GmbH) at a flow rate of 300 nL/min with the following gradient program: 0–2 min, 2% to 5% B; 2–44 min, 5% to 28% B; 44–51 min, 28% to 40% B; 51–53 min, 40% to 100% B; 53–60 min, 100% B. Following separation, peptides were analyzed using a Q-Exactive HF-X mass spectrometer (Thermo Scientific) operated in data-dependent acquisition (DDA) mode over a 60-min analysis time. MS analysis was performed in positive ion mode with full scans ranging from *m*/*z* 350 to 1800 at a resolution of 60,000 (at *m*/*z* 200), an AGC target of 3e6, and a maximum injection time of 50 ms. For MS/MS analysis, the 20 most intense precursors from each full scan were selected for fragmentation with the following parameters: resolution 15,000 (at *m*/*z* 200), AGC target 1 × 10^5^, maximum IT 50 ms, HCD activation, isolation window 1.6 *m*/*z*, and normalized collision energy 28. The raw data were processed using MaxQuant software (version 2.4.14.0) with specific search parameters detailed in Table 1. In the “Unique” column, “Yes” indicates that the peptide is unique, while “No” denotes a shared peptide.

#### 2.5.2. In Silico Screening of the Identified Peptides

Computer simulation is now a key tool widely used for screening and analyzing bioactive peptides [27]. The Peptide Ranker program (http://distilldeep.ucd.ie/PeptideRanker/ accessed on 29 October 2024) was chosen to predict the potential bioactivity of the identified peptide sequences, with higher scores indicating a higher likelihood of the sequence being biologically active. The potential toxicity of the identified peptide sequences was evaluated online using the Toxin Pred program (https://webs.iiitd.edu.in/raghava/toxinpred/ accessed on 29 October 2024) [28]. The key physicochemical properties of the peptides were predicted online using the PepDraw tool (http://www.tulane.edu/~biochem/WW/PepDraw/index.html accessed on 30 October 2024) and PepCalc software (http://pepcalc.com/ accessed on 30 October 2024). BIOPEP-UWM: Bioactive peptides (https://biochemia.uwm.edu.pl/biopep/peptide_data.php accessed on 30 October 2024) were used to predict whether the resulting peptides had been reported [29]. Finally, the antioxidant activity of the peptide sequences was predicted using the AnOxPePred−1.0 program [30] (https://services.healthtech.dtu.dk/services/AnOxPePred-1.0/ accessed on 30 October 2024), and peptide sequences with a threshold value > 0.5 were chosen for subsequent experiments.

### 2.6. Molecular Docking and Binding Mode Analysis

Peptide sequences (SDF format) and SOD protein structures (PDB ID: 3KBE) were obtained from PubChem and PDB databases, respectively. Water molecules and small-molecule ligands were removed using PyMol 2.1.0, hydrogenated and charged using AutoDock Tools 1.5.6, and saved in PDBQT format. Semi-flexible blind protein–peptide docking was performed using Vina 2.0 integrated in PyRx 0.9.9. The docking results were visualized using PyMol, and 2D interaction diagrams were generated by Discovery Studio 2020.

### 2.7. Molecular Dynamics (MD) Simulations

All-atom molecular dynamics simulations based on the peptide and SOD complexes obtained by 2.5 molecular docking were carried out as initial structures using AMBER 22 software [31]. The peptide charge was calculated by Gaussian 09 (HF/6-31G*) [32], and the peptide and SOD were described using the GAFF2 peptide force field and the ff14SB protein force field [33], respectively. The system was hydrogenated by the LEaP module and solvated in a 10 Å TIP3P aqueous cartridge [34] with Na^+^/Cl^−^ equilibrium charge.

Energy optimization (2500-step steepest descent method and 2500-step conjugate gradient method) was performed prior to the simulation, followed by gradual warming (0–298.15 K, 200 ps) and equilibration of the system under NVT (isothermal isobaric) (500 ps) and NPT (isothermal isobaric) (500 ps) tethering, and ultimately 100 ns NPT (isothermal isobaric) simulations. For the calculation of non-bonding interactions (including van der Waals forces and electrostatic interactions), a truncation distance of 10 Å was set, Particle mesh Ewald (PME) was used to calculate long-range electrostatic interactions [35], SHAKE to constrain hydrogen bonding [36]. Langevin temperature control [37], where the collision frequency γ was set to 2 ps^−1^, the system pressure to 1 atm, the integration step to 2 fs, and the trajectories were saved every 10 ps for subsequent analysis.

### 2.8. Statistical Analysis

Data were derived from at least three independent experiments and expressed as mean ± standard deviation (SD). Statistical significance was determined by one-way analysis of variance (ANOVA) followed by Duncan using IBM SPSS Statistics 26, with a significance threshold of *p* < 0.05. Graphical representations were generated using Origin 2022 software (OriginLab Corporation, Northampton, MA, USA).

## 3. Results

### 3.1. Antioxidant Activity of Sea Cucumber Gonadal Peptides and Fractionation

**Figure 1:**
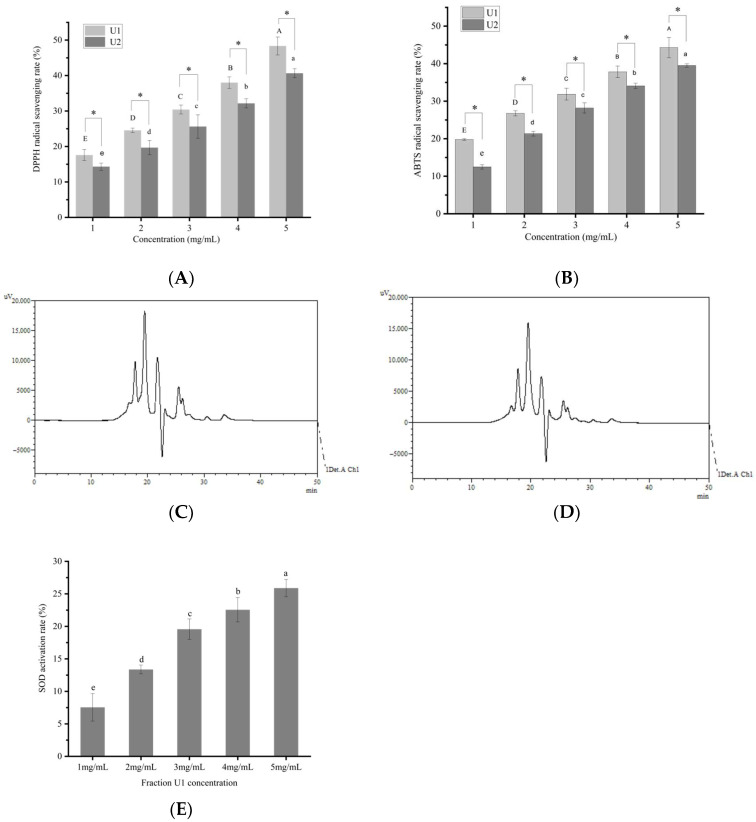
Antioxidant activity of different fractions. (**A**) Scavenging activity against 2,2-diphenyl-1-picrylhydrazyl (DPPH) free radicals. (**B**) Scavenging activity against 2,2′-azinobis(3-ethylbenzothiazoline-6-sulfonic acid) (ABTS) free radicals. (**C**) High-performance liquid chromatography (HPLC) chromatogram of fraction U1 (200–3000 Da). (**D**) HPLC chromatogram of fraction U2 (>3000 Da). (**E**) Activation rate of superoxide dismutase (SOD). Different capital or lowercase letters in the figure indicate statistically significant differences (*p* < 0.05). *, *p* < 0.05.

The assessment of in vitro antioxidant properties through DPPH and ABTS radical scavenging assays has been widely used. As shown in Figure 1A,B, at the concentration of 1–5 mg/mL, peptide fractions U1 and U2 exhibited a significant concentration-dependent increase (*p* < 0.05) in both DPPH and ABTS free radical scavenging activities. At 5 mg/mL, the DPPH free radical scavenging rates of U1 and U2 reached 48.3 ± 0.24% and 40.4 ± 0.25%, respectively, while their ABTS free radical scavenging activities were 44.4 ± 0.26% and 39.3 ± 0.20%, respectively. Notably, U1 demonstrated significantly higher antioxidant activity than U2 (*p* < 0.05). The distinct antioxidant activities exhibited by these two fractions may be related to their molecular weight (MW) distributions (Figure 1C–D). In fraction U1, peptides below 2000 Da accounted for 93.56%, with those below 1000 Da constituting 67.47%. In contrast, fraction U2 contained 63.77% peptides below 2000 Da and only 14.65% below 1000 Da (Table 2). Clearly, the proportion of peptides <1000 Da in U1 was significantly higher than in U2. It has been reported that the MW of marine-derived antioxidant peptides typically ranges between 370 and 3000 Da [38,39]. Thus, the higher antioxidant activity observed in fraction U1 is likely attributed to its greater content of lower-molecular-weight peptides.

### 3.2. SOD Activation Rate of U1 Fraction

SOD converts superoxide anions into molecular oxygen and hydrogen peroxide, thereby eliminating free radicals and maintaining redox homeostasis in organisms. It plays a pivotal role in the direct scavenging of free radicals in biological systems [40]. A significant concentration-dependent effect (*p* < 0.05) of SOD activation was observed (Figure 1E), enhancing approximately 25.90% of increase at 5 mg/mL of fraction U1. Currently, very few peptides were reported to bind to SOD to exert antioxidant effects. Reference [20] demonstrated that supplementation with the peptide FDGDF significantly enhanced SOD activity by 11.64% compared to the control group. The higher SOD activation rate suggested that peptide fraction U1 contained greater SOD-activating peptides.

### 3.3. Identification of Antioxidant Peptides in U1 Fraction and In Silico Screening

**Figure 2:**
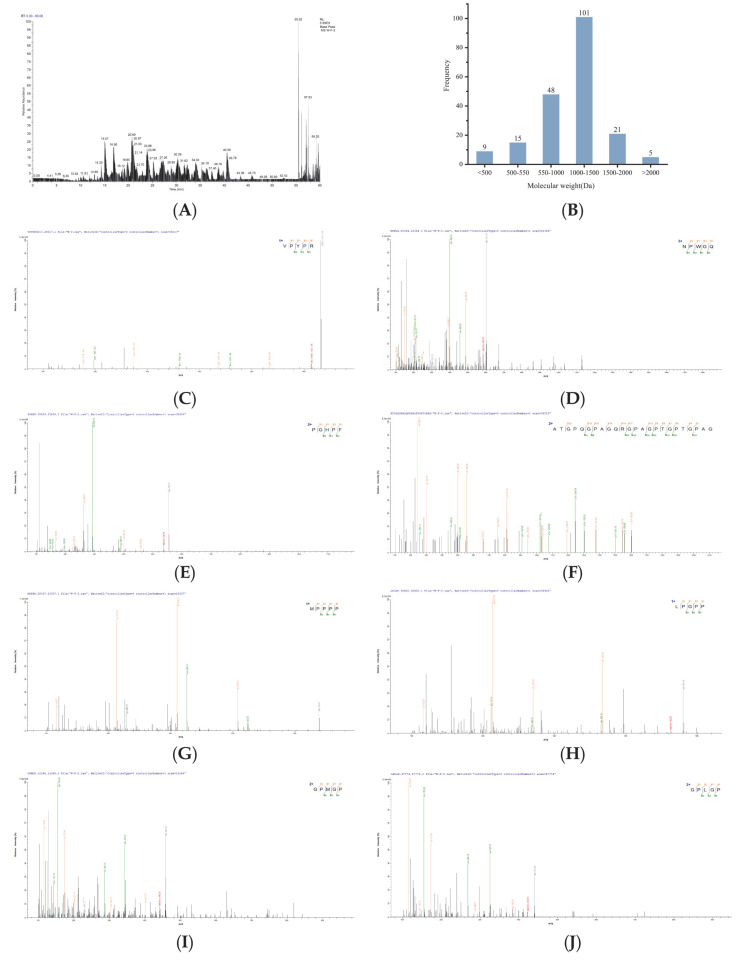
MS/MS spectra of identified peptides. (**A**) LC-MS/MS mass spectrum of the U1 fraction (200–3000 Da), molecular weight (**B**) and peptides (**C**) VPYPR, (**D**) NPWGQ, (**E**) PGHPF, (**F**) ATGPQGPAGQRGPAGPTGPTGPAG, (**G**) MPPPP, (**H**) LPGPP, (**I**) GPMGP, and (**J**) GPLGP.

Peptide component U1 was selected for peptide sequencing, leading to the identification of 199 peptide sequences (Table S1). The mass spectrometry profiles are displayed in Figure 2A, and the molecular weights of the peptide fragments are shown in Figure 2B. Bioinformatics approaches can facilitate the discovery of novel bioactive peptides from protein hydrolysates [41]. Thus, in silico tools were employed to predict and rank the potential bioactivity of these peptides. PeptideRanker results revealed that 46 peptide sequences exhibited scores exceeding 0.5, with higher scores indicating stronger bioactivity and reliability [42]. Further predictive analysis of toxicity, physicochemical properties, and antioxidant activity (Table 3) demonstrated that 8 peptide sequences reaching the threshold of both PeptideRanker score and radical scavenging capacity score above 0.5 [43] were predicted to be non-toxic. Their molecular weights ranged from 439.51 to 2057.18 Da. Studies indicated that most antioxidant peptides derived from food proteins exhibit molecular weights ranging from 500 to 1800 Da [44] and typically comprise 2 to 20 amino acid residues [45], which aligns with the present findings. The MS/MS spectra of the selected 8 peptides are presented in Figure 2C–J.

### 3.4. Molecular Docking of the Identified Peptides to SOD

**Figure 3:**
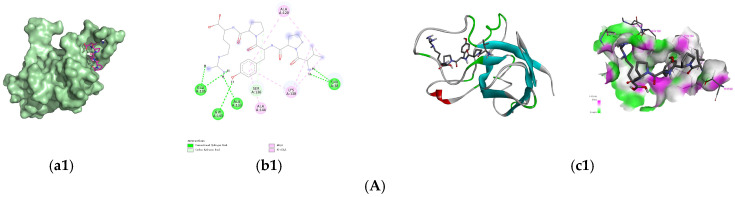

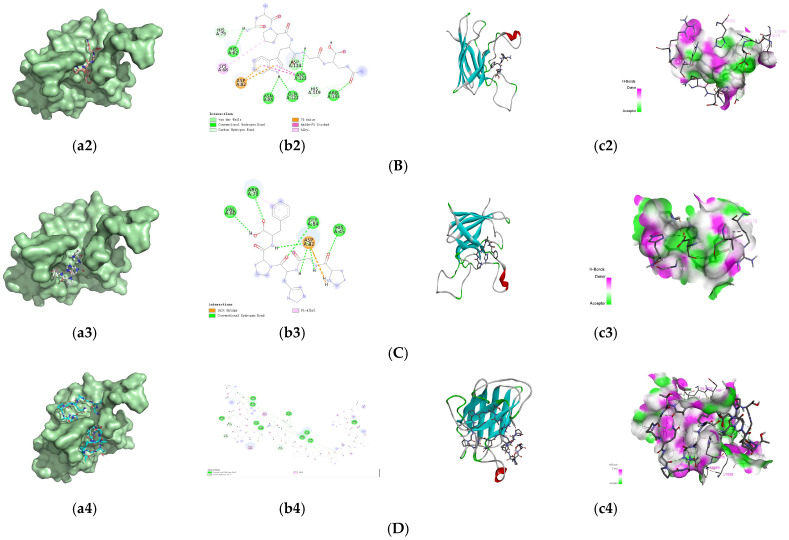
Predicted binding modes of the identified peptides to SOD. (**A**) VPYPR; (**B**) NPWGQ; (**C**) PGHPF; (**D**) ATGPQGPAGQRGPAGPTGPTGPAG. Each peptide binding to SOD was presented as (**a1**–**a4**) surface diagram, (**b1**–**b4**) 2D diagram binding residues, and (**c1**–**c4**) overall structure and local binding pocket diagram.

Since Figure 1E showed that peptide fraction U1 activated SOD, SOD was selected as the target for the screened antioxidant peptides to exert antioxidant activity; their binding energy of the screened 8 peptides to SOD are presented in Table 4. Generally, a docking energy value below −4.25 kcal/mol suggests mild binding activity between the molecules, below −5.0 kcal/mol indicates good binding activity, and below −7.0 kcal/mol signifies strong binding activity [46]. Among the nine peptides screened, four sequences, i.e., VPYPR, ATGPQGPAGQRGPAGPTGPTGPAG (AT-AG), PGHPF, and NPWGQ, exhibited free energies below −7.0 kcal/mol and were further selected as potential peptides binding to SOD. The docking modes and interactions between peptides and SOD were visualized using PyMol software, as shown in Figure 3.

The optimal conformation of VPYPR formed four conventional hydrogen bonds with residues Thr38, Glu135, Ala133, and Gly143, a carbon–hydrogen bond with Ser136, alkyl interactions with Ala120 and Lys138, and pi-alkyl interactions with Ala120, Lys137, Lys138, and Ala144. NPWGQ formed five conventional hydrogen bonds with residues His62, Asn85, Gln122, Asp123, and Arg145, two carbon–hydrogen bonds with His119 and His79, a pi-anion interaction with Asp82, an amide-pi stacked interaction with Asp123, and an alkyl interaction with Lys68. PGHPF formed four conventional hydrogen bonds with Val80, Arg78, Gly84, and His45, a salt bridge with Asp82, and a pi-alkyl interaction with Arg78. AT-AG formed eleven conventional hydrogen bonds with Gln48, Asn64, Lys68, Arg78, Asn85, Ala144, Arg145, Gln122, Asp123, Asp82, and Asn85, carbon–hydrogen bonds with His47, His79, and His119, an alkyl interaction with Pro61, and a pi-alkyl interaction with His70. In this study, residues such as Arg, Lys, Asp, and His frequently appeared, consistent with previous findings [47,48].

Generally, proteins and peptides interact through multiple forces, including hydrogen bonds, van der Waals forces, hydrophobic interactions, and electrostatic interactions, with hydrogen bonds and hydrophobic interactions being the key forces in peptide–SOD binding [49]. The docking results demonstrated that the four antioxidant peptides primarily bound to SOD via hydrogen bonds, electrostatic interactions, and hydrophobic interactions with specific amino acid residues, thereby enhancing SOD activation.

### 3.5. Molecular Dynamics Simulation of Peptide–SOD Binding

**Figure 4:**
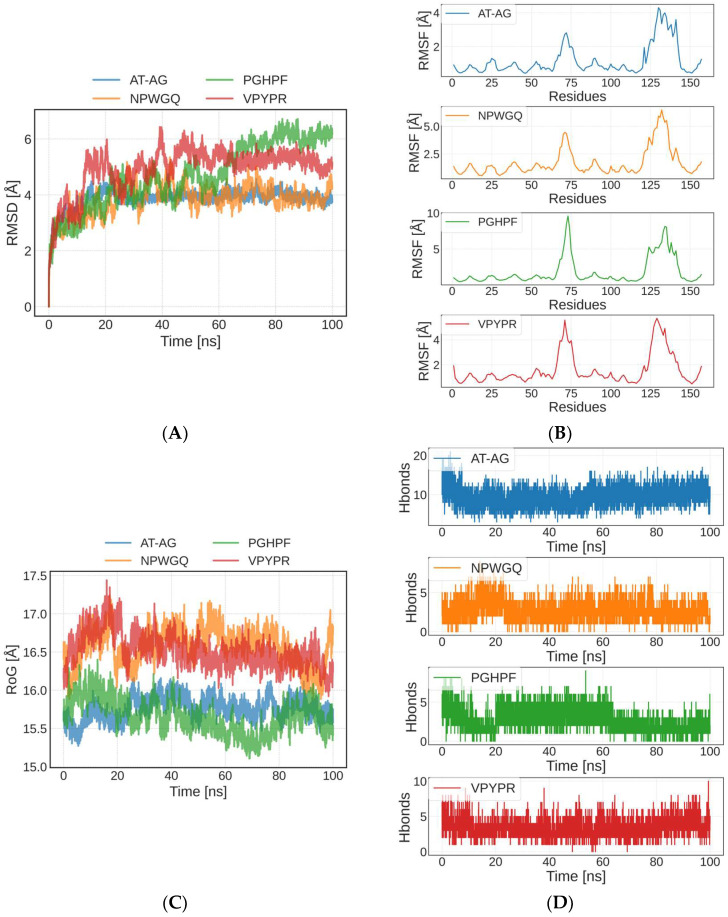
Molecular dynamics simulation of peptide–SOD complex. (**A**) The variation in Root Mean Square Deviation (RMSD). (**B**) The root-mean-square fluctuation (RMSF). (**C**) The variation in RoG. (**D**) The amount of hydrogen bonds.

Molecular dynamics simulation, a computer simulation method based on classical physics, has been widely adopted to investigate molecular interactions and structural dynamics at the atomic scale [50]. Using the initial conformation of a complex formed by four peptides with SOD, a molecular dynamics simulation in 100 ns was performed (Figure 4). The stability and flexibility of the receptor–ligand complexes were evaluated by analyzing the root-mean-square deviation (RMSD) and root-mean-square fluctuation (RMSF) of atomic coordinates, where increased deviations and fluctuations may indicate reduced stability [51]. RMSD measures the displacement of atoms relative to their initial conformation, and monitoring its changes helps reveal structural variations in protein complexes during simulations, thereby assessing their stability and dynamics [52]. As shown in Figure 4A, the AT-AG-SOD and NPWGQ-SOD complexes exhibited relatively stable overall fluctuations, with the AT-AG-SOD complex demonstrating particularly high stability. The low RMSD values (<10 Å) suggest minimal structural fluctuations during the simulation, strongly indicating that these two peptides maintained stable binding conformations within the SOD binding pocket [53]. Although the PGHPF-SOD and VPYPR-SOD complexes showed slightly higher RMSD values, they remained below 10 Å, confirming their high stability within the SOD binding site.

The RMSF is used to characterize the fluctuation degree of each atom or residue during the simulation [54]. A higher RMSF value reflects greater flexibility in the peptide structure. This adaptability facilitates conformational optimization, thereby enabling stable interactions with SOD [55] (Figure 4B). The four peptide–SOD complexes exhibited relatively stable trajectories throughout the simulation, with higher flexibility observed primarily near residue 75 and residues 125–150. Among them, the PGHPF-SOD complex displayed the largest RMSF values, which may be attributed to the fewer hydrogen bonds and other chemical interactions formed in this complex. These observations were consistent with the molecular docking results and the numbers of hydrogen bonds (Figure 4D) presented in Section 3.4.

The radius of gyration (RoG) represents the average distance from the molecular center of mass to all atomic positions, reflecting the overall compactness or expansion of the molecule [56]. A smaller RoG value indicates a more compact molecular structure, while a larger RoG suggests greater expansion. Throughout the simulation, the four peptide–SOD complexes exhibited minimal fluctuations in RoG, demonstrating overall structural stability (Figure 4C). These results further confirmed that the AT-AG, NPWGQ, PGHPF, and VPYPR peptides form tightly bound and stable complexes with SOD.

### 3.6. In Vitro Antioxidant Activity of the Screened Peptides

**Figure 5:**
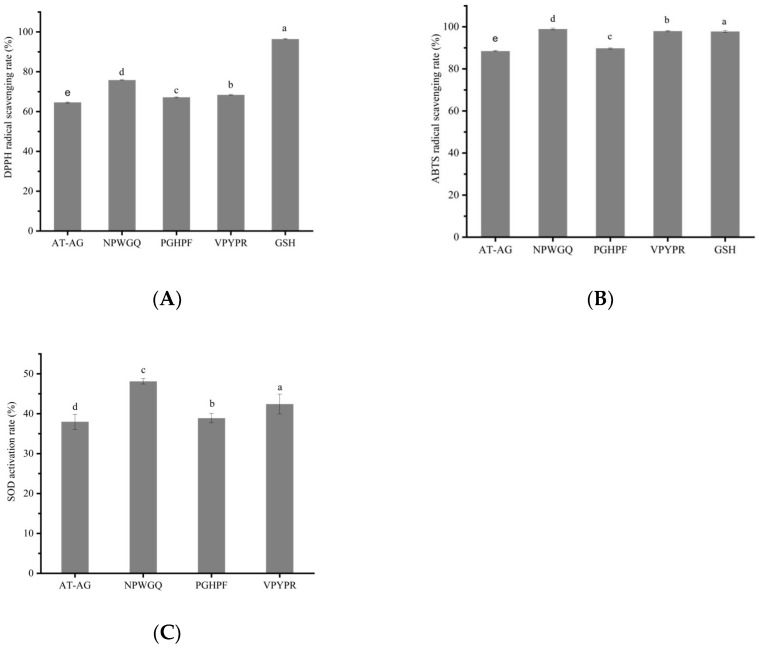
The validation of antioxidant activity of synthetic peptides. (**A**) DPPH free radical scavenging activity. (**B**) ABTS free radical scavenging activity. (**C**) SOD activation rate. Different letters among different peptide groups indicate statistically significant (*p* < 0.05).

Compared to the U1 component, the four novel antioxidant peptides exhibited significantly higher DPPH radical scavenging activity, ABTS cation radical scavenging activity, and SOD activation rate (*p* < 0.05) (Figure 5A–C). At a concentration of 5 mg/mL, the DPPH radical scavenging rates of VPYPR, NPWGQ, PGHPF, and AT-AG were 68.4 ± 0.23%, 75.8 ± 0.28%, 67.2 ± 0.23%, and 64.6 ± 0.29%, respectively, representing increases of 41.6%, 56.9%, 39.1%, and 33.7% compared to the U1 component. The scavenging capacity followed the order: NPWGQ > VPYPR > PGHPF > AT-AG. Compared to the plant-derived protein peptide MSePGP at 10 mg/mL, the scavenging rate of NPWGQ was 10.66% higher, while the activities of VPYPR, PGHPF, and AT-AG were comparable [57]. Relative to the stone fish peptide GVSGLHID at 5 mg/mL, the four peptides showed increases of 5.23%, 16.62%, and 3.38%, respectively, with AT-AG exhibiting similar scavenging capacity to the peptide GVSGLHID [58].

In the ABTS radical scavenging assay, at 5 mg/mL, the scavenging rates of the four peptides were 98.0 ± 0.30%, 98.9 ± 0.34%, 89.8 ± 0.32%, and 88.5 ± 0.32%, corresponding to improvements of 120.7%, 122.7%, 102.3%, and 99.3%, respectively. Notably, the ABTS scavenging capacities of VPYPR and NPWGQ were comparable to or even higher than that of the positive control GSH (*p* < 0.05). The scavenging capacity ranked as NPWGQ > VPYPR > PGHPF > AT-AG. Compared to the phycocyanin peptide at 5 mg/mL, the scavenging rates of the four peptides were 10.63%, 11.65%, and 1.38% higher, respectively, while AT-AG showed similar activity [59]. Relative to the stone fish peptide GVSGLHID, the four peptides demonstrated increases of 15.29%, 16.35%, 5.65%, and 4.12% [58].

In the SOD activation assay, at 5 mg/mL, the activation rates of the four peptides were 42.43 ± 2.51%, 48.12 ± 0.68%, 38.91 ± 1.18%, and 38.00 ± 1.89%, representing enhancements of 63.80%, 85.79%, 50.23%, and 46.72% compared to U1, respectively. The activity order remained NPWGQ > VPYPR > PGHPF > AT-AG. Thus, these peptides demonstrated consistent activity in DPPH and ABTS radical scavenging as well as SOD activation.

## 4. Discussion

This study systematically reveals, for the first time, the antioxidant properties and potential molecular mechanisms of sea cucumber gonad-derived peptides, addressing a critical gap in the research on bioactive peptides from key tissue sources for the high-value utilization of sea cucumber by-products. In contrast to the extensively studied body wall peptides of sea cucumber, the peptide composition and functional properties of sea cucumber gonads, as processing by-products, have long been underexplored [60]. Through in vitro antioxidant assays, this study confirms that sea cucumber gonad peptides exhibit significant activity in scavenging DPPH radicals and ABTS^+^ cations, showing comparable or even superior efficacy to the reported body wall peptide VLLYQDHCH, which demonstrated scavenging rates of 57.79 ± 0.83% and 88.39 ± 0.08%, respectively [61]. Further analysis of peptide composition via liquid chromatography–mass spectrometry revealed that gonad peptides possess unique amino acid sequence characteristics, including a high proportion of hydrophobic amino acids (such as tyrosine, proline, alanine, valine, glycine, and tryptophan) and multiple potential antioxidant short peptide sequences. Additionally, this study is the first to explore the interaction between sea cucumber gonad peptides and SOD. Preliminary results indicate that these peptides form strong bindings with SOD through hydrogen bonding, electrostatic interactions, and hydrophobic effects. These findings not only confirm sea cucumber gonads as a high-quality source of bioactive peptides but also highlight their uniqueness in structure–activity relationships, thereby expanding the theoretical boundaries for the diversified utilization of sea cucumber resources.

Free radical scavenging is one of the primary mechanism for peptides to exhibit antioxidant activity. NPWGQ exhibited the strongest antioxidant activity among the four peptides, followed by VPYPR, then PGHPF, with AT-AG showing the lowest activity. Peptides’ antioxidant activity is highly dependent on sequence and amino acid composition, wherein hydrophobic residues (Trp, Ala, Pro, Leu, Gly, Tyr, and Val) play a critical role in free radical scavenging [62,63,64]. This observation aligns with reports that hydrophobic residues constitute approximately 55% of antioxidant peptide sequences in conger eel [44]. Consistent with these findings, the hydrophobic amino acid content in VPYPR, NPWGQ, PGHPF, and AT-AG reached 80%, 60%, 80%, and 75%, respectively. The hydrophobic amino acids present in these four peptides include Tyr, Pro, Trp, Ala, Val, and Gly. Studies demonstrate that the phenolic hydroxyl group of tyrosine scavenges free radicals by donating hydrogen, forming more stable phenoxyl radicals that subsequently inhibit free radical-induced peroxidation chain reactions [63,65,66,67,68].The pyrrolidine ring of proline enhances peptide flexibility and quenches singlet oxygen due to its low ionization potential [69,70].The nitrogen–hydrogen bond in the indole ring of tryptophan serves as a critical hydrogen-donating site for radical scavenging, constituting a key mechanism for its antioxidant activity [63]. Additionally, Val and Ala residues at the N-terminus confer distinct antioxidant properties to the peptide sequences [71].

In vitro assays demonstrated that these four bioinformatically identified peptides exhibit antioxidant effects via dual mechanisms: direct radical scavenging and enhancement of superoxide dismutase (SOD) reductive activity. Superoxide dismutases (SODs), a class of highly conserved metalloenzymes, are classified into four major types—FeSOD, MnSOD, NiSOD, and Cu/ZnSOD—based on their metal cofactors and are widely distributed across bacteria, plants [72], microorganisms [73], and aquatic organisms [74]. Among these, Cu/ZnSOD plays a pivotal role in the first line of defense against oxidative stress by converting reactive oxygen species (ROS) into hydrogen peroxide, which is subsequently degraded to water by catalase. Recent advances in SOD activity modulation have focused on two primary strategies: (1) chemical modification to improve stability and delivery efficiency, and (2) screening natural bioactive compounds to directly enhance enzymatic activity.

In the context of chemical modification, conjugation of Cu/ZnSOD with O-(2-hydroxyl) propyl-3-trimethyl ammonium chitosan chloride (O-HTCC) yielded the O-HTCC-SOD complex. Although a partial reduction in β-sheet conformation led to an approximately 19% loss of initial activity, the complex exhibited significantly improved pharmacokinetics and membrane permeability due to its broadened molecular weight distribution and enhanced secondary structural ordering. Notably, the composite enabled controlled release of SOD activity through gradual degradation by α-amylase, ultimately achieving a 2.3-fold increase in catalytic efficiency [75]. In parallel, breakthroughs have been made in screening SOD activators from natural products in traditional Chinese medicine. Using UF-HPLC-Q-TOF-MS coupled with molecular docking, eight SOD ligands—including dibenzocyclooctene lignans and coumarins—were identified from Schisandra chinensis and Acanthopanax senticosus. These ligands uniquely enhanced SOD activity by reducing substrate–enzyme binding affinity while elevating catalytic turnover rates [76].

SOD activation assays revealed that the antioxidant peptides identified in this study scavenge free radicals through dual mechanisms: direct self-binding and, more notably, by targeting SOD to amplify its radical-eliminating activity. While numerous peptides enhance cellular SOD expression via NRF2/Keap1-dependent or independent pathways, few have been reported to directly bind and activate SOD. Notably, Ref.[20] isolated an antioxidant peptide, FDGDF, from dry-cured beef, which was shown to elevate SOD activity by stabilizing its β-sheet structure and modulating secondary conformation.

In FDGDF, the charge center of Phe5 forms a π-stacking interaction with Val113 of the SOD protein, while the carbonyl oxygen of Gly forms a hydrogen bond with Ser111. The amino group of Gly also forms a hydrogen bond with the backbone carbonyl oxygen of Arg108. Additionally, the two Asp residues form a salt bridge and hydrogen bonds with Arg108. As shown in Figure 3B(b1–b4), each amino acid in the peptide NPWGQ establishes chemical interactions with one or more residues in SOD. Specifically, the amide N-H of the Asn side chain forms a hydrogen bond with the Nδ or Nε of the imidazole ring of His62 in SOD. The Cδ-H_2_ of Pro engages in an alkyl–alkyl hydrophobic interaction with the Cγ-H_2_ of Lys68. Trp exhibits multiple interactions with SOD: its indole ring acts as a π-electron donor, forming a π-anion interaction with the carboxylate anion of Asp82, an amide-π stack with the -CONH_2_ group of amidated Asp123, and hydrogen bonds with the side-chain carbonyl oxygen of Asn85 and the amide group of Gln122. The backbone carbonyl oxygen of Gln forms a hydrogen bond with the backbone N-H of Arg145. Furthermore, van der Waals forces exist between Asn and His79, as well as between Gln and His119, while a C-H bond is observed between Trp and Asp124. Based on these findings, it can be inferred that the binding site of NPWGQ on SOD (involving His62, Lys68, Asp82, Asp123, Asn85, Gln122, and Arg145) may be a very promising binding pocket for discovering future peptides that activate SOD.

To investigate the actual sites of action of the antioxidant peptides (e.g., VPVPR, NPVVGQ) identified through computational simulation under physiological conditions, we focused on evaluating their ability to traverse the cell membrane, which is critical for determining their potential intracellular functionality [77]. Prediction results from the CellPPD tool indicated that all tested peptides were predicted as “non-cell-penetrating peptides.” Although some peptides (e.g., VPVPR) carry a positive charge, their overall structures lack typical cell-penetrating peptide (CPP) characteristics. Furthermore, the size of larger peptides (e.g., ATGPQQFAQQRGPAGPTGPTGPAG) also constitutes a physical barrier for passive transmembrane transport.

Based on these predictions, we infer that these antioxidant peptides are more likely to exert their functions primarily in the extracellular space. Their antioxidant mechanisms may include directly scavenging free radicals in the plasma or extracellular matrix [78] and protecting membrane-surface lipids and proteins from oxidative damage [79], rather than acting directly on intracellular targets. This understanding is crucial for accurately interpreting their potential physiological functions and application scenarios. In future studies, we will systematically validate this hypothesis through biochemical assays, cellular-level verification, and mechanistic investigations.

## 5. Conclusions

Four novel antioxidant peptides, VPYPR, NPWGQ, PGHPF, and ATGPQGPAGQRGPAGPTGPTGPAG, were identified from the sea cucumber gonad proteins. Compared with the U1 fraction, these peptides exhibited significantly enhanced scavenging capacities against DPPH and ABTS radicals, as well as SOD activation ability (*p* < 0.05). Notably, NPWGQ demonstrated the most potent antioxidant activity and SOD activation. Molecular interaction analysis revealed that the antioxidant peptides primarily bind to SOD residues through hydrogen bonds, electrostatic interactions, and hydrophobic interactions. Moreover, all four peptides formed stable binding conformations with SOD. Further studies will focus on their metabolic stability in vivo and the underlying signaling pathways to elucidate their antioxidant mechanisms including whether the peptides can also enhance SOD expression. This study provides a foundation for the utilization of sea cucumber gonad resources and offers theoretical insights for the discovery of natural and safe antioxidant peptides.

A major limitation of this study is that the activity evaluation was exclusively based on in vitro experiments. Although some peptides demonstrated excellent antioxidant activity in vitro, their actual efficacy in vivo remains uncertain. Key issues such as stability in the gastrointestinal environment, absorption efficiency after oral administration, actual activity in vivo, and potential synergistic or antagonistic interactions among peptide components require further validation. Therefore, we have prioritized the systematic assessment of the in vivo behavior and interactions of these peptides as a key focus for subsequent research. This will include simulated gastrointestinal digestion experiments, studies on cellular permeability, validation of synergistic effects in peptide combinations, functional evaluation in animal models, analysis of in vivo metabolic stability, and in-depth exploration of related signaling mechanisms. These investigations aim to comprehensively elucidate their antioxidant principles, including whether they possess the ability to promote SOD expression, and to further explore their scientific formulation and synergistic application strategies in functional foods. This will systematically reveal the actual efficacy in vivo, synergistic potential, and translational prospects of these peptides. The present study not only lays the foundation for the deep processing and high-value utilization of sea cucumber gonad resources but also provides an important theoretical basis for the discovery of natural, safe antioxidant peptides and the study of their synergistic mechanisms.

## Figures and Tables

**Table 1:** MaxQuant Analysis Parameter Settings.

Item	Value
Enzyme	Unspecific
Max Missed Cleavages	0
Precursor Tolerance (Main search)	4.5 ppm
Precursor Tolerance (First search)	20 ppm
MS/MS Tolerance	20 ppm
Fixed modifications	-
Variable modifications	Oxidation (M), Acetyl (Protein N-term)
Database	uniprot-Haliotis discus hannai (Japanese abalone) [42344]-314-20240620.fasta; uniprot-Apostichopus (genus) [307971]-30561- 20240620.fasta
Database pattern	Target-Reverse
PSM FDR	0.01
Protein FDR	0.01
Site FDR	0.01

**Table 2:** Molecular weight distribution of peptide fraction from sea cucumber gonad.

Molecular Weight Range (Da)	Proportion of Fraction U1 (%)	Proportion of Fraction U2 (%)
>2000	6.439	36.233
1000~2000	26.090	49.122
500~1000	60.615	10.247
200~500	6.856	4.398

**Table 3:** Predicted ranking of antioxidant peptide activity from sea cucumber gonad.

Peptide	PeptideRanker Score	Toxicity	Mass	PL	Free Radical Scavenging Ability
VPYPR	0.641232	Non-toxic	630.74	9.81	0.55691272
ATGPQGPAGQRGPAGPTGPTGPAG	0.562816	Non-toxic	2057.18	10.9	0.54368335
PGHPF	0.946647	Non-toxic	553.61	8.26	0.58514124
NPWGQ	0.832775	Non-toxic	600.62	3.28	0.53533584
MPPPP	0.959959	Non-toxic	537.67	3.57	0.57264793
GPMGP	0.929226	Non-toxic	457.55	3.82	0.52304268
GPLGP	0.841507	Non-toxic	439.51	3.82	0.50645763
LPGPP	0.887134	Non-toxic	479.57	3.82	0.54216856

**Table 4:** Binding affinity of peptides from sea cucumber gonad.

Peptide	Binding Affinity (kcal/mol)
VPYPR	−7.6
ATGPQGPAGQRGPAGPTGPTGPAG	−7.5
PGHPF	−7.4
NPWGQ	−7.2
MPPPP	−6.7
GPMGP	−6.4
GPLGP	−6.4
LPGPP	−6.2

## Data Availability

The original contributions presented in this study are included in the article. Further inquiries can be directed to the corresponding author. The mass spectrometry proteomics data have been deposited in the ProteomeXchange Consortium (https://proteomecentral.proteomexchange.org accessed on 27 October 2025)) via the iProX partner repository with the dataset identifier PXD069933.

## Supplementary Materials

The following supporting information can be downloaded at: https://www.mdpi.com/article/10.3390/foods14223848/s1, Table S1. LC-MS/MS analysis of 199 identified gonad peptides from sea cucumber.
