# Supplementary material for: Screening and Identification of Antioxidant Peptides from Sea Cucumber Gonad Proteins and Their Activation of Superoxide Dismutase
Source: Foods. 2025 Nov 11;14(22):3848. doi: 10.3390/foods14223848 (PMC12651066; doi:10.3390/foods14223848)
Supplement: Supplementary file 1 [file foods-14-03848-s001.zip › foods-3919122-supplementary.pdf]

Table S1. LC-MS/MS analysis of 199 identified gonad peptides from sea cucumber.

Sequence	Length	Mass（Da）	Proteins	Leading razor protein	Unique	Score	PSM
AVSEGTKAVTKYTTSK	16	1669.8887	A0A2G8KT35;A0A2G8KT32	A0A2G8KT35	yes	266.75	2
AGPTGPTGP	9	753.36572	A0A1I9W676	A0A1I9W676	yes	236.77	1
VGAQGERGEAGNTGPQ	16	1526.7073	A0A1I9W676	A0A1I9W676	no	229.56	1
GPAGPTGPTGPA	12	978.47706	A0A1I9W676	A0A1I9W676	yes	227.3	1
TNWDDMEK	8	1037.4124	C5NTJ9;A0A2G8JRZ2;A0A2G8KMT7;A0A2G8JBD9;A0A2G8KJ01;A0A2G8JSN3;A0A2G8K0Q5	C5NTJ9	no	226.55	1
GPTGPTGPA	9	753.36572	A0A1I9W676	A0A1I9W676	yes	224.39	1
NPDHPIIE	8	933.4556	R4HC40;G4XU66;A0A097GVX9;A0A6M2VKR8;A0A2G8JL82	R4HC40	yes	221.25	1
AGPTGPTGPAG	11	881.4243	A0A1I9W676	A0A1I9W676	yes	219.7	1
NEATGGKYVPR	11	1190.6044	A0A2G8KRV2;A0A2G8KRX3;A0A2G8KYX9;A0A2G8KRX5;A0A2G8JR39	A0A2G8KRV2	yes	217.5	1
AVSEGTKAVTKYTTS	15	1541.7937	A0A2G8KT35;A0A2G8KT32	A0A2G8KT35	yes	215.34	2
DLKNKANEEVEK	12	1415.7256	A0A2G8K7R8	A0A2G8K7R8	yes	214.86	1
LNDTKGSSGVAIKK	14	1416.7936	A0A2G8LB13;A0A2G8JRI4;A0A2G8K942;A0A2G8JX95;A0A2G8K918	A0A2G8LB13	yes	213.99	2
VGEGMEEGE	9	935.35423	A0A2G8JN23;A0A2G8K0Q6;A0A2G8K0S7;A0A2G8JYC3;A0A2G8K0R4;A0A0M4ATJ8;A0A2G8JIJ4;A0A2G8LFU4;A0A2G8K0U2;A0A2G8K0R2;A0A2G8LLY4;A0A2G8LPX8;A0A2G8LLX1;A0A2G8JQV5;A0A2G8LFY3;A0A2G8K0P7	A0A2G8JN23	no	200.66	1
SEGTKAVTKYTTS	13	1371.6882	A0A2G8KT35;A0A2G8KT32	A0A2G8KT35	yes	198.57	2

AIADLNDPKGSSGTAIKK	18	1784.9632	A0A2G8JDJ2;A0A2G8LCY5	A0A2G8JDJ2	yes	189.09	3
VTEDEGNEQTDGRP	14	1545.6543	A0A2G8JTU0	A0A2G8JTU0	yes	188.61	1
GVETGVLK	8	801.45962	A0A2G8JLY2;A0A2G8JDJ2;A0A2G8LCY5	A0A2G8JLY2	no	186.43	1
GAAGAAGAAGSQGNQGER	18	1528.6978	A0A2G8LKB5	A0A2G8LKB5	yes	186.17	1
GATGERGPAGPS	12	1055.4996	A0A2G8LKB8	A0A2G8LKB8	yes	185.6	1
SKPTGPTT	8	787.40758	A0A2G8LB13;A0A2G8JRI4;A0A2G8K942;A0A2G8JX95;A0A2G8K918	A0A2G8LB13	no	183.9	1
AGPTGPTGPA	10	824.40283	A0A1I9W676	A0A1I9W676	yes	180.18	1
AGDSKKDPPIQAE	13	1354.6729	A0A2G8JU26;A0A2G8L9Y8	A0A2G8JU26	yes	179.19	1
IPLPEEN	7	810.41233	A0A2G8JKY2	A0A2G8JKY2	yes	178.65	1
GGGTGSGMGTL	11	893.39128	A0A2G8KRV2;A0A2G8KRX3;A0A2G8KYX9;A0A2G8KS26;A0A2G8KRV0	A0A2G8KRV2	yes	168.66	1
TPTYGDL	7	765.35448	A0A2G8KRV2;A0A2G8KRX3;A0A2G8KS26;A0A2G8KRV0;A0A2G8JQW5	A0A2G8KRV2	yes	167.23	1
AGDSKKDPPIQAET	14	1455.7205	A0A2G8JU26;A0A2G8L9Y8	A0A2G8JU26	yes	165.39	1
TPTYGDLNH	9	1016.4563	A0A2G8KRV2;A0A2G8KRX3;A0A2G8KS26;A0A2G8KRV0;A0A2G8JQW5	A0A2G8KRV2	yes	164.53	1
ETPIYEQ	7	878.40216	A0A2G8K7R8	A0A2G8K7R8	yes	163.84	1
GQDGEDGRDGAQGPA	15	1428.5866	A0A1I9W676	A0A1I9W676	yes	163.45	1
GPANPDNIPEDGR	13	1350.6164	A0A2G8LQ00;A0A2G8LQ99	A0A2G8LQ00	yes	162.38	1
TGPAGPQGPAGDRGQ	15	1364.6433	A0A1I9W676	A0A1I9W676	no	160.22	1
DKEGIPPDQQR	11	1281.6313	A0A2G8LBR5;A0A2G8KUQ6;A0A2G8KDT4;A0A2G8KC12;A0A2G8KC39;A0A2G8KDU5;A0A2G8J	A0A2G8LBR5	yes	159.83	1

			G69;A0A2G8JG54;A0A2G8JG56;A0A2G8JG89;A0A2G8JG81				
FGDDISQDNEDNR	13	1523.6124	E3WHU2	E3WHU2	yes	159.09	1
AAGAAGAAGSQGNQGERGRQGAQG	24	2125.9962	A0A2G8LKB5	A0A2G8LKB5	yes	159.04	1
GQRGPAGPTGPTGP	14	1248.6211	A0A1I9W676	A0A1I9W676	yes	158.94	1
GPAGPQGP	8	679.32894	A0A1I9W676	A0A1I9W676	no	158.16	1
GVATGVLK	8	743.45414	A0A2G8LB13;A0A2G8JRI4;A0A2G8K942;A0A2G8JX95;A0A2G8K918	A0A2G8LB13	yes	158.12	1
APVISAEKAYH	11	1184.619	A0A2G8JN23;A0A2G8K0Q6;A0A2G8K0S7;A0A2G8K0Q9;A0A2G8JYC3;A0A2G8JIJ4;A0A2G8JQX3;A0A2G8LFU4	A0A2G8JN23	no	157.96	1
IPPGGTEP	8	766.38612	A0A2G8LH72	A0A2G8LH72	yes	156.93	1
ISKEEYDESGPS	12	1339.578	C5NTJ9;A0A2G8JRZ2	C5NTJ9	yes	156.16	1
AVANAQDFSHQ	11	1186.5367	A0A2G8KWZ0;A0A2G8LM02	A0A2G8KWZ0	yes	155.58	1
HKDTPENNPNTP	12	1362.6164	A0A2G8LRY6	A0A2G8LRY6	yes	150.27	1
DNYPRDGKHPTD	12	1413.6273	A0A2G8LAG4	A0A2G8LAG4	yes	149.96	1
ATGPQGPAGQRGPAGPTGPTGPA	23	1998.9872	A0A1I9W676	A0A1I9W676	yes	149.59	1
IDPTGTYHGD	10	1074.4618	A0A2G8KRV2;A0A2G8KRX3;A0A2G8KYX9;A0A2G8KRX5;A0A2G8JR39	A0A2G8KRV2	yes	149.4	1
LPFQR	5	659.37549	A0A2G8KMC0;A0A2G8LB53;A0A1S6YDW3;A0A2G8K8I7;A0A2G8LN81;A0A2G8K0X6	A0A2G8KMC0	no	145.19	2
SPAPGSEKSRAGQP	14	1367.6793	A0A2G8KGP3	A0A2G8KGP3	yes	143.97	2
ISGTSMATPH	10	1000.4648	W6FIM7;Q1WDP1;A0A2G8K8I3;A0A2G8K9E4	W6FIM7	yes	143.85	1
REEDIPPGADGR	12	1310.6215	A0A2G8L9T0	A0A2G8L9T0	yes	142.19	2

FDDNIGDHE	9	1060.4098	A0A2G8KWZ0;A0A2G8LM02	A0A2G8KWZ0	yes	141.39	1
NRKSTITSREVQT	13	1518.8114	A0A2G8KT35;A0A2G8KT32	A0A2G8KT35	yes	141.37	2
IDGVEGRQGPA	11	1097.5465	A0A2G8LKB5	A0A2G8LKB5	yes	140.83	1
GQRGPAGPTGPTGPAGA	17	1447.7168	A0A1I9W676	A0A1I9W676	yes	140.14	1
GQRGPAGPTGP	11	993.49919	A0A1I9W676	A0A1I9W676	yes	138.26	1
EGPQGPNGERGPTGD	15	1466.6386	A0A3G1ZIU9;A0A2G8LQN2	A0A3G1ZIU9	yes	138.24	1
GQRGPAGPTGPTGPAG	16	1376.6797	A0A1I9W676	A0A1I9W676	yes	137.55	1
AQGERGEAGNTGPQ	14	1370.6175	A0A1I9W676	A0A1I9W676	no	136.81	1
GYRGSQGERGPYG	13	1382.6327	A0A2G8LKB5	A0A2G8LKB5	yes	136.5	1
LNDTKGSSGVAIK	13	1288.6987	A0A2G8LB13;A0A2G8JRI4;A0A2G8K942;A0A2G8JX95;A0A2G8K918	A0A2G8LB13	yes	135.26	1
TGVDNPGHPF	10	1039.4723	A0A2G8LAG4	A0A2G8LAG4	yes	135	1
HGPTAPFEGNKEA	13	1353.6313	A0A2G8LFZ4;A0A2G8JEV0	A0A2G8LFZ4	yes	134.57	1
PGGDLAKVQR	10	1039.5774	A0A2G8JN23;A0A2G8K0Q6;A0A2G8K0S7;A0A2G8K0R1;A0A2G8JYC3;A0A2G8K0R4;A0A0M4ATJ8;A0A2G8K0R2;A0A2G8LPX8;A0A2G8LLX1;A0A2G8JQV5	A0A2G8JN23	no	133.32	1
VPGTGTDGSGLGHES	15	1369.611	A0A2G8KMF0	A0A2G8KMF0	yes	132.39	1
QVHPDTGISSRAMS	14	1484.7042	A0A2G8KT35;A0A2G8KT32	A0A2G8KT35	yes	131.82	2
YVGDEAQSKRG	11	1208.5786	C5NTJ9;A0A2G8JRZ2;A0A2G8KMT7;A0A2G8JBD9;A0A2G8KJ01;A0A2G8JSN3;A0A2G8K0Q5	C5NTJ9	no	131.82	2
GATGERGPSGP	11	984.46247	A0A1I9W676	A0A1I9W676	yes	131.52	1
VPGTGTDGSGLGHESG	16	1426.6325	A0A2G8KMF0	A0A2G8KMF0	yes	131.46	1
AVSEGTKAVTKY	12	1252.6663	A0A2G8KT35;A0A2G8KT32	A0A2G8KT35	yes	131.42	1
KQVHPDTGISSRAMS	15	1612.7991	A0A2G8KT35;A0A2G8KT32	A0A2G8KT35	yes	130.72	2

LPVLD	5	555.32681	A0A2G8JUA3	A0A2G8JUA3	no	129.54	1
SYVGDEAQSKRG	12	1295.6106	C5NTJ9;A0A2G8JRZ2;A0A2G8KMT7;A0A2G8JBD9;A0A2G8KJ01;A0A2G8JSN3;A0A2G8K0Q5	C5NTJ9	no	128.52	1
QRGPAGPTGPTGPA	14	1262.6367	A0A1I9W676	A0A1I9W676	yes	128.27	1
ATGPQGQQGSRGERGPEGQQG	21	2080.9635	A0A1I9W676	A0A1I9W676	no	127.32	1
GQRGPAGPTGPT	12	1094.5469	A0A1I9W676	A0A1I9W676	yes	125.97	1
KDTGSPIRIP	10	1082.6084	A0A2G8JRT9	A0A2G8JRT9	yes	125.75	1
FDGDVGDQG	9	908.35119	A0A2G8LKB5	A0A2G8LKB5	yes	125.53	1
EEGEF	5	609.22822	A0A2G8JN23;A0A2G8K0Q6;A0A2G8K0S7;A0A2G8JYC3;A0A2G8K0R4;A0A0M4ATJ8;A0A2G8JIJ4;A0A2G8LFU4;A0A2G8K0U2;A0A2G8K0R2;A0A2G8LLY4;A0A2G8LPX8;A0A2G8LLX1;A0A2G8JQV5;A0A2G8K0P7;A0A2G8JDR7;A0A2G8JN38;A0A2G8LQ66;A0A2G8KD09;A0A2G8L2J3;A0A2G8K0U6;A0A2G8LH39;A0A2G8LH66;A0A2G8JKJ9;A0A2G8JQ73;A0A2G8KRL6;A0A2G8LHA5;A0A2G8KRV2;A0A2G8KRX3;A0A2G8KS26;A0A2G8JQW5;A0A2G8KRV7	A0A2G8KRV2	no	124.87	1
GQRGPAGPTGPTGPAGATGERGPSGPQ	27	2414.1687	A0A1I9W676	A0A1I9W676	yes	124.51	1
AVSEGTKAVTK	11	1089.603	A0A2G8KT35;A0A2G8KT32	A0A2G8KT35	yes	123.96	2
VPFPR	5	614.35403	A0A2G8KRV2;A0A2G8KRX3;A0A2G8KYX9;A0A2G8KS26;A0A2G8KRV0;A0A2G8JQW5;A0A2G8KRV7;A0A2G8JDG4;A0A2G8K9U2	A0A2G8KRV2	no	123.86	1

ATGPQGPAGQRGPAGPTGPTGPAG	24	2056.0086	A0A1I9W676	A0A1I9W676	yes	122.93	1
HSLGGGTGSGMGT	13	1117.4822	A0A2G8KRV2;A0A2G8KRX3;A0A2G8KYX9;A0A2G8KS26;A0A2G8KRV0	A0A2G8KRV2	yes	122.28	1
IGGSSNNDSGKVD	13	1248.5582	A0A2G8LNP3	A0A2G8LNP3	yes	121.96	1
DKEGIPPDQQRL	12	1394.7154	A0A2G8LBR5;A0A2G8KUQ6;A0A2G8KDT4;A0A2G8KC12;A0A2G8KC39;A0A2G8KDU5;A0A2G8JG69;A0A2G8JG54;A0A2G8JG56;A0A2G8JG89	A0A2G8LBR5	yes	121.6	2
GSTGPAGPQGPAGDR	15	1323.6167	A0A1I9W676	A0A1I9W676	no	121.02	1
LPVNE	5	570.30133	E3WHU2;A0A2G8JZP6;A0A2G8JGB7;A0A2G8LP07;A0A2G8JSY8;A0A2G8JFX5;A0A2G8JHV2;A0A2G8JL41	E3WHU2	no	120.06	1
SKLEF	5	622.33263	A0A2G8JN23;A0A2G8K0Q6;A0A2G8K0S7;A0A2G8K0R1;A0A2G8K0Q9;A0A2G8JYC3;A0A2G8K0R4;A0A2G8JIJ4;A0A2G8JQX3;A0A2G8K0R2;A0A2G8LLY4;A0A2G8JWX4;A0A2G8KQS3;A0A2G8JEJ7;A0A2G8KTS4	A0A2G8JN23	no	120.06	2
APLNP	5	510.2802	C5NTJ9;A0A2G8JRZ2;A0A2G8KMT7;A0A2G8KMS5;A0A8G1M2U6;A0A2G8LMH4;A0A2G8L5J3;A0A2G8KSZ9;A0A2G8LCM7;A0A2G8KDB9;A0A2G8LAD7;A0A2G8K9B4;A0A2G8JJD8;A0A2G8JHC0;A0A2G8L932;A0A2G8JH86;A0A2G8K937;A0A2G8LEF4	C5NTJ9	no	120	1
GPFGQ	5	504.23325	A0A2G8KRV2;A0A2G8KRX3;A0A2G8KYX9;A0A2G8KS26;A0A2G8KRV0;A0A2G8KRX5;A0A2G8KRV7;A0A2G8JR39	A0A2G8KRV2	no	120	1

KQVHPDTGISSRAM	14	1525.7671	A0A2G8KT35;A0A2G8KT32	A0A2G8KT35	yes	119.45	2
AVSEGTKAVTKYT	13	1353.714	A0A2G8KT35;A0A2G8KT32	A0A2G8KT35	yes	119.03	1
LPGEL	5	527.29551	A0A2G8KT35;A0A2G8KT32;A0A2G8LJV4;A0A2G8KQV0;A0A2G8LCG8;A0A2G8KKV2	A0A2G8KT35	no	116.54	1
TGEGMDEME	9	997.33686	A0A2G8KRV2;A0A2G8KRX3;A0A2G8KYX9;A0A2G8KS26;A0A2G8KRV0;A0A2G8JQW5;A0A2G8KRV7	A0A2G8KRV2	yes	116.54	1
GVATGVLKQ	9	871.51272	A0A2G8LB13;A0A2G8JRI4;A0A2G8K942;A0A2G8JX95;A0A2G8K918	A0A2G8LB13	yes	116.3	1
SEGTKAVTKYTTSK	14	1499.7831	A0A2G8KT35;A0A2G8KT32	A0A2G8KT35	yes	114.69	1
AAEASRLAHY	10	1087.5411	A0A2G8KT35;A0A2G8KT32	A0A2G8KT35	yes	113.74	1
SKPTGPTTL	9	900.49165	A0A2G8LB13;A0A2G8JRI4;A0A2G8K942;A0A2G8JX95;A0A2G8K918	A0A2G8LB13	no	113.5	1
YVPPAEDSEEQR	12	1418.6314	A0A2G8KGP3	A0A2G8KGP3	yes	112.75	1
NPWGQ	5	600.26561	A0A2G8KKC3	A0A2G8KKC3	no	112.58	1
VPVVN	5	526.3115	A0A2G8JTU0;A0A2G8JRH1;A0A2G8LE08;A0A2G8JQF7;A0A1W5LDR6;A0A2G8K9S1	A0A2G8JTU0	no	112.31	1
STGPAGPQGPAGDRGQ	16	1451.6753	A0A1I9W676	A0A1I9W676	no	111.96	1
GHQGPRGDQGEDGADGRDGA	20	1950.8165	A0A2G8LKB5	A0A2G8LKB5	yes	111.95	2
AVSEGTKAVTKYTT	14	1454.7617	A0A2G8KT35;A0A2G8KT32	A0A2G8KT35	yes	111.94	1
IGGIGTVPVGR	11	1024.6029	A0A2G8JU26;A0A2G8L9Y8	A0A2G8JU26	yes	110.87	1
VAGDSKKDPPIQAET	15	1554.789	A0A2G8JU26;A0A2G8L9Y8	A0A2G8JU26	yes	110.08	1
RGTPGGPDKKGEAGAGSTDN	20	1870.8769	A0A2G8KKS8;A0A2G8JEL8	A0A2G8KKS8	yes	109.66	1
ATGERGPSGPQ	11	1055.4996	A0A1I9W676	A0A1I9W676	yes	109.02	1

GPAGPQGEVGDR	12	1138.5367	A0A1I9W676	A0A1I9W676	no	108.43	1
SVGESGSRGPAGA	13	1130.5316	A0A2G8LKB5	A0A2G8LKB5	yes	107.79	1
REEDIPPGADGRR	13	1466.7226	A0A2G8L9T0	A0A2G8L9T0	yes	106.32	2
YNEATGGKYVPR	12	1353.6677	A0A2G8KRV2;A0A2G8KRX3;A0A2G8KYX9;A0A2G8KRX5;A0A2G8JR39	A0A2G8KRV2	yes	106.29	2
SETGAGKHVPRA	12	1208.6262	A0A2G8JN23;A0A2G8K0Q6;A0A2G8K0S7;A0A2G8K0R1;A0A2G8K0Q9	A0A2G8JN23	no	105.4	1
VTYAP	5	549.27986	A0A2G8JN23;A0A0M4ATJ8;A0A2G8JQX3;A0A2G8LFU4;A0A2G8K0U2	A0A2G8JN23	yes	105.36	1
SEGPF	5	535.22783	A0A2G8K7R8;A0A2G8LKH2;A0A2G8KXX8;A0A2G8LR75	A0A2G8K7R8	no	104.62	1
MPPPP	5	537.2621	A0A2G8LAJ9;A0A2G8K445;A0A2G8KTY1;A0A2G8KFI6;A0A2G8JUN5;A0A2G8KTW3;A0A2G8JS09;A0A2G8JYC4;A0A2G8LGP3;A0A2G8KQX3;A0A2G8L9W2;A0A2G8JZX7;A0A2G8L5Q0;A0A2G8LFY0;A0A2G8JZH9;A0A2G8JDW3;A0A2G8LB29;A0A2G8K151;A0A2G8JBS4	A0A2G8LAJ9	no	104.08	1
PKGSSGTAIKK	11	1072.6241	A0A2G8JDJ2;A0A2G8LCY5	A0A2G8JDJ2	yes	103.91	1
REEDIPPGADGRRE	14	1595.7652	A0A2G8L9T0	A0A2G8L9T0	yes	103.3	1
VPYPR	5	630.34895	A0A2G8JN23;A0A2G8K0Q6;A0A2G8K0S7;A0A2G8K0R1;A0A2G8K0Q9;A0A2G8JYC3;A0A2G8K0R4;A0A0M4ATJ8;A0A2G8JIJ4;A0A2G8JQX3;A0A2G8LFU4;A0A2G8K0U2;A0A2G8LLY4;A0A2G8LLW7;A0A2G8LFY3;A0A2G8JKV4;A0A2G8LMB9;A0A2G8JFJ9;A0A2G8JQ21;A0A2G8JIA4;A0A2G8JN15;A0A2G8LPI8	A0A2G8JN23	no	102.87	2

IDPTGTYHGDSD	12	1276.5208	A0A2G8KRV2;A0A2G8KRX3;A0A2G8KYX9;A0A2G8KRX5;A0A2G8JR39	A0A2G8KRV2	yes	101.64	1
KQVHPDTGISSR	12	1323.6895	A0A2G8KT35;A0A2G8KT32	A0A2G8KT35	yes	101.39	1
ATGPQGPAGQRGPA	14	1263.632	A0A1I9W676	A0A1I9W676	yes	99.864	1
ALKRGVETGVLK	12	1269.7769	A0A2G8JLY2;A0A2G8JDJ2;A0A2G8LCY5	A0A2G8JLY2	no	99.788	2
FPGQL	5	560.29585	A0A2G8KRV2;A0A2G8KRX3;A0A2G8KYX9;A0A2G8KS26;A0A2G8KRV0;A0A2G8JQW5;A0A2G8KRV7;A0A2G8LAW6;A0A2G8KFM9;A0A2G8KAU0	A0A2G8KRV2	no	99.626	1
GPVQY	5	562.27511	A0A2G8LKB8;A0A2G8L3B4;A0A2G8KJU7	A0A2G8LKB8	no	99.605	1
PERKY	5	691.36532	C5NTJ9;A0A2G8JRZ2;A0A2G8KMT7;A0A2G8KMS5;A0A8G1M2U6;A0A2G8JBD5;A0A2G8KJ01;A0A2G8JSN3	C5NTJ9	no	97.926	1
APMNP	5	528.23662	A0A2G8KJ01;A0A2G8JSN3;A0A2G8K0Q5;A0A2G8K6W8;A0A2G8L0H8	A0A2G8KJ01	no	97.866	2
SDYNIQKESTLH	12	1433.6787	A0A2G8LBR5;A0A2G8KUQ6;A0A2G8KDT4;A0A2G8KC12;A0A2G8KC39;A0A2G8KDU5	A0A2G8LBR5	yes	96.342	1
EHPVL	5	593.31731	C5NTJ9;A0A2G8JRZ2;A0A2G8KMT7;A0A2G8KMS5;A0A8G1M2U6;A0A2G8L5J3;A0A2G8LCI1;A0A2G8KJ01;A0A2G8JSN3	C5NTJ9	no	94.533	1
ATGPQGQQGSRGERGPEGQQGQAG	24	2337.0806	A0A1I9W676	A0A1I9W676	no	94.359	1
IAALNDSKGSSGSAIKK	17	1645.8999	A0A2G8JLY2	A0A2G8JLY2	yes	94.203	1
IDERGPIPTDRR	12	1423.7532	A0A2G8JRT9	A0A2G8JRT9	yes	93.494	1
PSNLGTGLR	9	913.49813	A0A2G8LAG4;A0A2G8LAS7;A0A2G8LAG8;A0A2G8JGI3	A0A2G8LAG4	yes	93.374	1

IADLNDPKGSSGTAIKK	17	1713.9261	A0A2G8JDJ2;A0A2G8LCY5	A0A2G8JDJ2	yes	93.152	1
LELPEDEEEKKK	12	1485.7563	R4HC40;G4XU66;A0A097GVX9;A0A6M2VKR8;A0A2G8JL82;D0V3X9	R4HC40	yes	91.961	1
GERGEAGNTGPQ	12	1171.5218	A0A1I9W676	A0A1I9W676	no	90.827	1
VAGTP	5	443.238	A0A3G1ZIU9;A0A2G8JPE5	A0A3G1ZIU9	no	90	1
VVPGGDLAKVQR	12	1237.7143	A0A2G8JN23;A0A2G8K0Q6;A0A2G8K0S7;A0A2G8K0R1;A0A2G8JYC3;A0A2G8K0R4;A0A0M4ATJ8;A0A2G8K0R2;A0A2G8LPX8;A0A2G8LLX1	A0A2G8JN23	yes	89.403	1
LPGPP	5	479.27438	A0A1I9W676;A0A2G8LCI5;A0A2G8JJU8;A0A2G8LI61;A0A2G8JW32;A0A2G8LQX1;A0A2G8JL05;A0A2G8LR12	A0A1I9W676	no	88.98	1
GEVGDRGNPGPA	12	1124.521	A0A1I9W676	A0A1I9W676	no	88.496	1
GDSKKDPPIQAET	13	1384.6834	A0A2G8JU26;A0A2G8L9Y8	A0A2G8JU26	yes	88.187	1
YNEATGGKYVPRA	13	1424.7048	A0A2G8KRV2;A0A2G8KRX3;A0A2G8KYX9;A0A2G8KRX5;A0A2G8JR39	A0A2G8KRV2	yes	88.134	1
NEATGGKYVPRA	12	1261.6415	A0A2G8KRV2;A0A2G8KRX3;A0A2G8KYX9;A0A2G8KRX5;A0A2G8JR39	A0A2G8KRV2	yes	86.898	1
GATGERGPSGPQ	12	1112.521	A0A1I9W676	A0A1I9W676	yes	86.879	1
LPLQD	5	584.31698	A0A2G8JU26;A0A2G8L9Y8;A0A2G8KNN3;A0A2G8L2W7;A0A2G8JV31	A0A2G8JU26	no	86.344	1
RPKDPADNEAG	11	1168.5473	E3WHU2	E3WHU2	yes	85.731	1
SILTTHTTLEHS	12	1338.6779	A0A2G8JN23;A0A2G8K0Q6;A0A2G8K0S7;A0A2G8K0R1;A0A2G8K0R4	A0A2G8JN23	yes	85.676	1
GQQGSRGERGPEGQQGQAG	19	1882.863	A0A1I9W676	A0A1I9W676	no	85.362	1
SGVERSSVFINK	12	1321.699	A0A2G8JLY2	A0A2G8JLY2	yes	85.355	1

VGESGSRGPAGA	12	1043.4996	A0A2G8LKB5	A0A2G8LKB5	yes	83.647	1
MESAGIHETTYN	12	1351.5714	C5NTJ9;A0A2G8JRZ2;A0A2G8JBD5;A0A2G8KJ01;A0A2G8JSN3	C5NTJ9	no	82.749	1
WAKGHYTEGAE	11	1247.5571	A0A2G8KRV2;A0A2G8KRX3;A0A2G8KYX9;A0A2G8KS26;A0A2G8KRV0;A0A2G8KRX5;A0A2G8KRV7	A0A2G8KRV2	yes	81.972	1
RKSTITSREVQT	12	1404.7685	A0A2G8KT35;A0A2G8KT32	A0A2G8KT35	yes	81.565	1
YRPGT	5	592.29691	A0A2G8KMC0;A0A2G8LB53;A0A1S6YDW3	A0A2G8KMC0	no	80.317	1
QVHPDTGISSRA	12	1266.6317	A0A2G8KT35;A0A2G8KT32	A0A2G8KT35	yes	77.662	1
VAGDSKKDPPIQA	13	1324.6987	A0A2G8JU26;A0A2G8L9Y8	A0A2G8JU26	yes	77.288	1
APLLL	5	525.35263	A0A2G8KKS8;A0A2G8K958;A0A2G8L3H9;A0A2G8JI08;A0A2G8KLQ3;A0A2G8KN52;A0A2G8L3K6;A0A2G8JEY4;A0A2G8JRJ7;A0A2G8JIW4;A0A2G8KU50	A0A2G8KKS8	no	76.733	1
VGYNP	5	548.25946	A0A2G8JU26;A0A2G8L9Y8	A0A2G8JU26	no	76.287	1
YEPTP	5	605.26969	E3WHU2;A0A2G8JZP6;A0A2G8LL60;A0A2G8JCR5;A0A2G8K5N6;A0A2G8L302	E3WHU2	no	76.053	1
DLVVK	5	572.35336	A0A2G8LB13;A0A2G8JRI4;A0A2G8K942;A0A2G8JX95;A0A2G8K918;A0A2G8LNI6;A0A2G8JLG1;A0A2G8JIF0;A0A2G8KHE9;A0A2G8JKD4;A0A2G8KAA0;A0A2G8L9R0;A0A2G8KA91;A0A2G8LJE6;A0A2G8JLY2	A0A2G8LB13	no	74.849	2
DMKVF	5	638.30978	A0A2G8K7R8;A0A2G8JE68;A0A2G8LAH5;A0A2G8JIT3	A0A2G8K7R8	no	74.849	1
GNQGPRGGPGETGK	14	1310.6327	A0A2G8LKB5	A0A2G8LKB5	yes	74.76	1

SAGIHETTYNS	11	1178.5204	C5NTJ9;A0A2G8JRZ2;A0A2G8JBD5;A0A2G8KJ01;A0A2G8JSN3	C5NTJ9	no	74.301	1
AATAAPQPGGPQ	12	1064.5251	A0A2G8LAJ9	A0A2G8LAJ9	yes	74.267	1
LNDPKGSSGTAIKK	14	1414.778	A0A2G8JDJ2;A0A2G8LCY5	A0A2G8JDJ2	yes	73.927	1
QPDGQMPSDKT	11	1202.5238	A0A2G8JN23;A0A2G8K0Q6;A0A2G8K0S7;A0A2G8K0R1;A0A2G8K0Q9;A0A0M4ATJ8;A0A2G8LLW7	A0A2G8JN23	no	73.885	1
FSETGAGKHVPR	12	1284.6575	A0A2G8JN23;A0A2G8K0Q6;A0A2G8K0S7;A0A2G8K0R1;A0A2G8K0Q9	A0A2G8JN23	no	73.848	1
GPDASEGDRSRGGGPGR	17	1626.7459	A0A2G8KJU7	A0A2G8KJU7	yes	73.802	1
PGHPF	5	553.26488	A0A2G8LAG4	A0A2G8LAG4	yes	73.666	1
LGGPL	5	455.27438	A0A2G8K8A9;A0A2G8JGL5	A0A2G8K8A9	no	73.666	1
GPLGP	5	439.24308	A0A1I9W676;A0A2G8JXZ2;A0A2G8L1E9;A0A2G8JL57	A0A1I9W676	no	73.666	1
SETGAGKHVPR	11	1137.5891	A0A2G8JN23;A0A2G8K0Q6;A0A2G8K0S7;A0A2G8K0R1;A0A2G8K0Q9	A0A2G8JN23	no	73.499	1
LPLKL	5	582.41048	A0A2G8K8A9;A0A2G8LG37;A0A2G8L771;A0A2G8KA51;A0A2G8LLS2;A0A2G8KIC2;A0A2G8KWY8;A0A2G8KG43;A0A2G8JS13	A0A2G8K8A9	no	73.289	2
GPMGP	5	457.1995	A0A3G1ZIU9;A0A2G8KE87;A0A2G8L2U7;A0A2G8LAJ5;A0A2G8L2V7;A0A2G8K7R0;A0A2G8L2V6;A0A2G8LBW2;A0A2G8KN86;A0A2G8L2W8;A0A2G8KWA5;A0A2G8KEA4;A0A2G8JCM0;A0A2G8LJG5;A0A2G8LI68;A0A2G8JH64	A0A3G1ZIU9	no	72.087	1

GPVGL	5	441.25873	A0A1I9W676;A0A2G8LGT0;A0A2G8KUP4;A0A2G8KUH5;A0A2G8KBY4;A0A2G8JQQ4;A0A2G8L6W1;A0A146AXJ2;A0A2G8LKB5	A0A1I9W676	no	72.087	1
VPLGM	5	515.27775	A0A2G8K8A9;A0A2G8K250	A0A2G8K8A9	no	72.087	1
KQVHPDTGISSRA	13	1394.7266	A0A2G8KT35;A0A2G8KT32	A0A2G8KT35	yes	71.879	1
EPGTM	5	533.21555	A0A2G8KRV2;A0A2G8KRX3;A0A2G8KYX9;A0A2G8KRV0;A0A2G8KRX5;A0A2G8JR39	A0A2G8KRV2	no	71.475	1
VHPDTGISSRAM	12	1269.6136	A0A2G8KT35;A0A2G8KT32	A0A2G8KT35	yes	71.349	1
GPDGQAGERGPRGPQ	15	1477.7022	A0A2G8LKB5	A0A2G8LKB5	yes	71.279	1
NKANEEVEKMK	11	1318.6551	A0A2G8K7R8	A0A2G8K7R8	yes	71.085	1
GPRGF	5	532.27578	A0A1I9W676;A0A2G8LDT5;A0A2G8LAM5;A0A2G8K9L0;A0A2G8LNE5;A0A2I4JGM2;A0A2G8JV51;A0A2G8KPD1;A0A2G8JD78;A0A2G8JDP7	A0A1I9W676	no	71.054	1
ERLLK	5	657.41736	A0A2G8LAG4;A0A2G8JI79;A0A2G8KLE2;A0A2G8L8Y8;A0A2G8KEY4;A0A2G8JQU4;A0A2G8L8M6;A0A7T1TTB6;A0A2G8KVJ4;A0A2G8K7Z7;A0A2G8KZU6;A0A2G8LRY4;A0A2G8LP48;A0A2G8JQR2;A0A2G8KXX9;A0A2G8KJQ1;A0A2G8LN27;A0A2G8LEG7;A0A2G8JB60;A0A6M2VJT4;A0A2G8K9M7;A0A2G8JKQ2;A0A2G8LBF6;A0A2G8LJG9	A0A2G8LAG4	no	69.39	1
YAPVISAEKAYH	12	1347.6823	A0A2G8JN23;A0A2G8K0Q6;A0A2G8K0S7;A0A2G8K0Q9;A0A2G8JYC3;A0A2G8JIJ4;A0A2G8JQX3;A0A2G8LFU4	A0A2G8JN23	no	69.276	1
FDGPEGPRGPPGSEGRQG	18	1795.8238	A0A1I9W676	A0A1I9W676	no	69.188	1

GDRGF	5	550.24996	A0A3G1ZIU9;A0A2G8L191;A0A2G8LI18	A0A3G1ZIU9	no	67.76	1
VTVLE	5	559.32173	A0A2G8JJL6	A0A2G8JJL6	no	67.327	1
PLTGP	5	483.2693	A0A2G8K7R8;A0A2G8KQ67	A0A2G8K7R8	no	66.538	1
LELPEDEEEKKKREE	15	1899.9426	R4HC40;G4XU66;A0A097GVX9;A0A6M2VKR8;A0A2G8JL82	R4HC40	yes	64.52	1
TYRYL	5	714.37008	A0A2G8K7R8;A0A2G8JFE3;A0A2G8KWH9;A0A2G8JT85;A0A2G8JIT7;A0A2G8JJC4	A0A2G8K7R8	yes	64.081	1
TGPDGQAGERGPR	13	1296.6171	A0A2G8LKB5	A0A2G8LKB5	yes	63.662	1
SPVLD	5	529.27478	A0A2G8JU26;A0A2G8L9Y8;A0A2G8JYA9;A0A2G8JSJ9;S6BN65;A0A2G8L870;A0A2G8KEA0;A0A2G8LEC8;A0A2G8JT78;A0A2G8KMJ9;A0A2G8K110;A0A2G8JXL2	A0A2G8JU26	no	63.581	1
DFKTE	5	638.29116	A0A2G8KMC0;A0A2G8LB53;A0A1S6YDW3;A0A2G8KP28;A0A2G8KGA7;A0A2G8LNX0;A0A2G8L343	A0A2G8KMC0	no	63.443	1
LNDSKGSSGSAIKK	14	1390.7416	A0A2G8JLY2	A0A2G8JLY2	yes	62.042	1
LPVQP	5	552.32715	A0A2G8KDD2;A0A2G8JJD0	A0A2G8KDD2	no	61.26	1
VGNLP	5	498.2802	A0A2G8KJU7	A0A2G8KJU7	no	61.26	1
VGSLL	5	487.3006	A0A2G8K8A9;A0A2G8JCN3;A0A2G8KMQ9;A0A2G8L7X7;A0A2G8L1T3;A0A2G8KB84;A0A2G8LPT8;A0A2G8KGG6;A0A2G8KIB5;A0A2G8L1K2;A0A2G8LRP0;A0A2G8JLB7;A0A2G8JR63;A0A2G8LHB9;A0A2G8LK16;A0A2G8JJ76;A0A2G8JDC1;A0A2G8LQY5;A0A2G8KMC7;A0A2G8LEA6;A0A2G8KNK7;A0A2G8KNK6	A0A2G8K8A9	no	60.874	1

VPGGDLAKVQR	11	1138.6459	A0A2G8JN23;A0A2G8K0Q6;A0A2G8K0S7;A0A2G8K0R1;A0A2G8JYC3;A0A2G8K0R4;A0A0M4ATJ8;A0A2G8K0R2;A0A2G8LPX8;A0A2G8LLX1;A0A2G8JQV5	A0A2G8JN23	no	60.255	1
TQQMF	5	653.2843	A0A2G8KRV2;A0A2G8KRX3;A0A2G8KYX9;A0A2G8KS26;A0A2G8KRV0;A0A2G8JQW5;A0A2G8KNV3;A0A2G8LAE6	A0A2G8KRV2	yes	59.539	1
VAAVF	5	505.29003	A0A2G8KRV2;A0A2G8LF82;A0A2G8K9G2;A0A2G8KAM9;A0A2G8KB76;A0A2G8KDM5;A0A2G8L1F7	A0A2G8KRV2	no	59.298	1
